# A comprehensive study on the effect of hybridization and stacking sequence in fabricating cotton-blended jute and pineapple leaf fibre biocomposites

**DOI:** 10.1016/j.heliyon.2023.e19792

**Published:** 2023-09-07

**Authors:** Tajwar A. Baigh, Fairooz Nanzeeba, Hasibur R. Hamim, M. Ahsan Habib

**Affiliations:** Department of Mechanical and Production Engineering, Islamic University of Technology (IUT), Gazipur, 1704, Bangladesh

**Keywords:** Natural fibre reinforced composites, Mechanical characterization, Moisture absorption, Vibration characteristics, Vacuum bagging, Applications

## Abstract

Developing biocomposites by hybridization, which is the combination of two or more materials, can be a potential solution for improving material recyclability and sustainability. This study focuses on creating a hybrid biocomposite reinforced with cotton-blended pineapple leaf fibre (PALF) fabric (174 GSM) and jute fibre fabric (265 GSM) which are thrown away by textile factories. The mechanical, moisture absorption, and vibration characteristics of four stacking sequences of hybrid composites and two unhybridized composites were analyzed. Results indicated that hybridization improved tensile and flexural characteristics compared to pineapple leaf fibre reinforced polymer (PFRP) composites. The jute fibre reinforced polymer (JFRP) composite exhibited the maximum tensile strength of 35.16 MPa, while the hybrid composites achieved a maximum of 32.16 MPa. Among the hybrid composites, jute layers on the outer plies (4P5J-2) demonstrated the maximum tensile modulus of 1.315 GPa. Additionally, the hybrid composite with three layers of jute plies between alternating layers of jute-pineapple plies showed the highest elongation at 15.94%. Among the hybrids, alternate stacking of jute/PALF plies (4P5J-1) gave a maximum flexural strength of 44.36 MPa, which is similar to JFRP (44.91 MPa) and a 78.57% increase in flexural modulus compared to PFRP composite, despite having the lowest tensile strength. Although the JFRP composite exhibited the highest impact strength, the hybrids still outperformed the PFRP composites. With hybridization, moisture absorption decreased, with a maximum of 29.50% compared to the JFRP composite. Furthermore, due to the spiral-like orientation of the yarns, stacking PALF plies on the outside can cause critical damping. Therefore, it is shown in this paper that both hybridization and stacking sequence can significantly influence composite performance. These findings also implies the utilization of textile industry's natural fibres to develop hybrid composites for automotive applications, like brake and accelerator pedals, for a greener future and effective waste material utilization.

## 1 Introduction

**Fig. 1:**
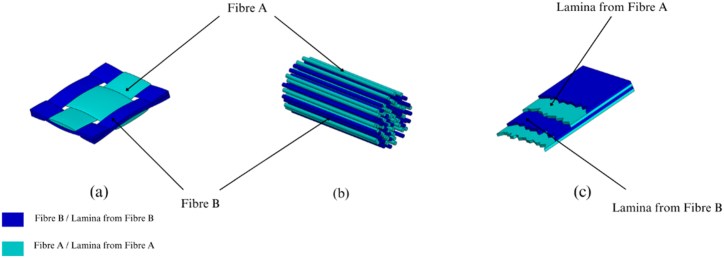
Different hybridization strategies of NFRCs: **(a)** Using threads/yarns of different origin for weaving the fabric; **(b)** Using yarns of multiple origins for constructing a hybrid fibre; **(c)** Stacking fabric and/or fibre laminas in any combination and orientation [2,3].

Over the past half-century, fibre-reinforced polymer (FRP) structural composites have gained priority over monolithic materials due to their unique benefits, including a favorable ratio of strength to weight and the ability to resist corrosion. Structural composites are arranged in a laminar form, with layers stacked on top of each other [1] (as shown in Fig. 1(a–c)).

Composites reinforced with synthetic fibres suffer from several significant drawbacks, such as (i) non-renewability, (ii) non - biodegradability, (iii) inability to be recycled, (iv) health hazards during the manufacturing process, and (v) significant energy consumption during fabrication. Recently, there has been a growing global emphasis on environmental awareness, leading to an increased focus on the development of recyclable and environmentally sustainable materials known as biocomposites [4]. Biocomposites are composites in which either the reinforcing material or matrix is derived from natural sources. Even though natural materials have poor mechanical properties, they can sometimes exhibit exceptional properties and unique functions due to their distinctive microstructure [5]. Additionally, agricultural and forest waste (approximately 30–40%) can also be utilized to create various value-added products [6].

Natural fibres can be commonly understood as a composite material consisting of three parts: cellulose, hemicellulose, and lignin. The natural fibre, also called a macrofibre is made up of numerous microfibrils which acts as the reinforcing materials in a matrix of hemicellulose and lignin [7]. As of the end of November 2022, the production of natural fibres, estimated at 32.2 million tonnes, remained largely unchanged from 2021 [8]. Natural plant fibres are frequently utilized in the production of biocomposites due to their affordability, strength, lightweight nature, high specific strength, moduli, superior thermal and acoustic insulation properties, and biodegradability [[9], [10], [11], [12]]. In fact, certain natural fibres can even rival the mechanical attributes of glass fibres [13,14]. In recent years, natural plant fibre-reinforced composites (NFRCs) have gained increasing popularity as structural composites due to their environmentally friendly characteristics and low-density properties [15]. It is predicted that the market for natural fibre-reinforced composites will reach $10.89 billion by 2024 [16] with a compound annual growth rate (CAGR) of 11.8% [17].

To compete with glass fibre composites, biocomposites need to undergo improvements, which often require costly surface alterations [18,19]. Consequently, hybridization has emerged as a popular technique in polymer technology. This approach enables the design of products with enhanced mechanical properties while mitigating issues related to moisture absorption and inadequate fibre/matrix bonding. Hybridization involves reinforcing with two different natural fibres or a combination of natural and synthetic fibres [[20], [21], [22]]. Excellent reinforcement can be achieved by hybridizing natural fibres such as jute, pineapple leaf fibre, sisal, hemp, banana, and oil palm fibre with glass fibre [[23], [24], [25], [26], [27]]. Compared to other lignocellulosic fibres, both PALF and jute contains a high amount of stiff natural cellulose [28] and low microfibrillar angle (helix angle between the cellulose fibrils and the axis of the secondary cell wall [29]), which results in superior mechanical properties when compared to many natural and synthetic fibres [[30], [31], [32]]. Table 1 provides a comparison of the physical properties of jute, PALF, and other commonly used natural fibres as reinforcement materials in fabricating NFRCs, while Table 2 offers a comparison of their chemical properties.

**Table 1:** Physical properties of natural fibres [12,19,21,32,[48], [49], [50], [51], [52], [53], [54]].

Fibre name	Fibre length (cm)	Density (g/cm³)	Tensile strength (MPa)	Specific tensile strength (MPa/g cm^−3^)	Elongation at break (%)	Young's modulus (GPa)	Specific young's modulus (GPa/g cm^−3^)	Moisture absorption (wt%)	Degree of crystallinity (X-ray)
Abaca	–	1.5	400	–	3–10	12	–	15	–
Bamboo	0.15–0.4	0.6–1.1	800–1200	–	10–20	15–20	25	8.9	–
Bagasse	1–30	1.25	290	–	1.1	17	18	8.8	–
Coir	0.8–33.7	1.2–1.32	95–230	110–180	15–37	4–6	3.3–5	10	25–33
Cotton	1–6	1.5–1.6	287–597	190–530	3–10	5.5–12.6	3.7–8.4	8–25	–
Flax	.5–90	1.4–1.5	345–1500	230–1220	1.2–3.2	27.6–80	18–53	7	44
Hemp	.5–5.5	1.5	550–900	370–740	1.6	70	39–47	8	44
**Jute**	**.2**–**12**	**1.1**–**1.34**	**393**–**800**	**300**–**610**	**1.5**–**1.8**	**10**–**78**	**7.1**–**39**	**12**	**52**–**60**
Kenaf	–	1.5	223–930	–	1.5–2.7	15–53	24	–	–
**Pineapple**	**90**–**150**	**0.8**–**1.6**	**180**–**1627**	**225**–**1016.9**	**1.6**–**14.5**	**1.44**–**82.5**	**35**	**13**	**—**
Sisal	90	1.33–1.5	507–855	362–610	2–2.5	9.4–28	6.7–20	11	–
Silk	Continuous	1.3	100–1500	100–1500	15–60	5–25	4–20	–	–

**Table 2:** Chemical composition of natural fibres [12,19,21,32,[48], [49], [50], [51], [52],^54^].

Fibre name	Cellulose (%)	Lignin (%)	Hemicellulose (%)	Pectin (%)	Ash (%)	Moisture content (wt %)	Waxes (%)	Microfibrillar Angle °
Abaca	56–63	7–9	15–17	1	0.5	5–10	3	–
Bamboo	26–43	25.3	16.8	–	–	–	–	–
Bagasse	55.2	25.3	16.8	–	–	–	–	–
Coir	36–43	40–45	0.15–24	3.3–4	–	8	–	30–49
Cotton	83–99	<2	5.7	0–1	–	7.85–8.5	0.6	–
Flax	71	2.2	18.6–20.6	2.3	–	8–12	1.7	5–10
Hemp	57–77	3.7–13	14–22.4	0.9	0.8	6.2–12	0.8	2–6.2
**Jute**	**61**–**71.5**	**12**–**26**	**13.6**–**21**	**0.2**	**0.5**–**2**	**12.5**–**13.7**	**0.5**	**8**
Kenaf	31–57	15–19	21.5–23	3–5	2–5	–	–	–
**Pineapple**	**81**	**12.7**	**—**	**—**	**—**	**14**	**—**	**14**
Sisal	47–78	7–11	10–14.2	10	0.6–1	10–22	2	10–22

Pineapple leaf fibres (PALF), are obtained from pineapple leaves, which are a type of natural fibre classified as leaf fibres [33]. Considering the significant annual production of approximately 13 million tonnes of pineapple agriculture waste, which includes PALF, proper management of the post-harvest waste is crucial [34]. PALF is highly valued in the textile industry due to its abundant availability, affordability, as well as good thermal properties and acoustic insulation, impressive tensile strength, and toughness [34]. It also has high onset oxidation temperatures of 240–272 °C, making it a good choice for most commercial polymers [31]. For hybrid composites incorporating PALF, improved tensile strength, flexural strength and impact strength have been observed in PALF-coir fibre hybridization in epoxy matrix [35]. Hybridization of PALF-kenaf in phenolic resin has shown improved interfacial strength and tensile, flexural and impact properties [36].

Jute plants have a remarkable environmental benefit, absorbing 15 tonnes of carbon dioxide (CO_2_) and releasing 11 tonnes of oxygen (O_2_) during their 120-day lifespan [37]. This high potential for carbon footprint reduction makes jute an excellent candidate for incorporation into structural composites. Life-cycle evaluation research comparing jute fibre-reinforced polymer (JFRP) composites with glass fibre-reinforced composites has shown improved environmental performance [38]. The use of jute in thermoset matrices, such as epoxy, has demonstrated superior tensile strength and flexural strength when compared to pure epoxy matrices [39], indicating its potential as a replacement for conventional structures. Previous studies commonly involved fabricating composites with jute and epoxy matrix [40], as well as jute in polyester matrix [41]. Jute-polyester composites with a 30% volume fraction have shown promise as replacements for wood and thermal insulation materials [41]. Jute-reinforced thermoset composites find applications in various structures and automotive interiors [42,43]. Long jute fibre (LFT) reinforced thermoplastic composites, particularly LFT polypropylene composites, are widely recognized as structural materials with favorable mechanical properties [44]. Other studies have analyzed jute/plastic composites, focusing on properties like thermal stability, crystallinity, and eco-design [38,[45], [46], [47]].

One problematic factor for natural fibres like jute and PALF is the poor adhesion between the fibre-matrix interface and hydrophobic thermosetting polymers, which results in inadequate stress transfer between the fibre and matrix. This impedes full utilization of the fibre strength, thus limiting the scope and scale of application and reducing fatigue life [55]. Chemical methods are the widely-used and the most convenient method of improving interface properties of organic fibre-based bio-composites [56]. Alkali treatment (mercerization) is one such method where, the treatment delignifies the natural fibre. It reduces the hydroxyl group on the natural fibre, effectively decreasing the hydrophilicity and roughens up the surface [57]. This can lead to the overall improvement in mechanical and moisture absorption performance to the composites fabricated with mercerized natural fibres.

Blending, which is the combination of different textile fibres, is a common practice employed to achieve fabrics with desirable properties [58]. Natural fibres and their blends with conventional fibres offer valuable characteristics, leading to the production of various fibre-based products. Blended yarns, compared to traditional yarns, exhibit improved moisture absorption and discharge, non-irritating and antibacterial properties, protection against harmful UV rays, and other valuable qualities [59]. Fabrics made from blended yarns often possess superior characteristics compared to those made from a single fibre [60]. Although PALF demonstrates excellent physical properties, its larger diameter makes it unsuitable for producing woven yarn. To address this, blending finer fibres such as cotton can be a solution, as it provides a higher fibre count per section [6]. Blending jute with cotton fibre can serve as an effective method for diversifying jute and creating value-added products. Jute fibres offer advantages such as a lustrous golden appearance, high tenacity, and good properties. Therefore, blending and softening techniques can be utilized to enhance the quality of jute and create a new class of jute-based fabrics with a growing market both domestically and internationally [61]. A study on the blending of jute with cotton revealed that the characteristics of blended yarn fabric can be equivalent to fully cotton fabric, potentially reducing dependence on imported cotton fibre. Thus, blending jute with cotton can help reduce the reliance on 100% cotton yarn [61]. Another study indicated that the thermal conductivity of a composite made from a blend of jute and cotton increased with the number of layers, except for the 50/50 jute-cotton combination. This suggests the potential use of jute-cotton blends as thermal insulation materials with high structural properties [42].

This study presents a unique application of abundant or wasted natural fibres in the textile industry by fabricating hybrid biocomposites. Jute fibre, PALF and cotton are derived from the stem, leaves and seeds of their respective plant genus. Hence, these three fibres have different morphological, chemical and mechanical properties. Jute is one of the stiffest lignocellulosic fibres and PALF is mechanically superior among leaf fibres [31]. The combination of these textile materials, which are from a variety of natural sources, to create structural biocomposites elucidates its potential for achieving sustainability while enhancing material recyclability and expands the applications of these waste textile fabrics. The hybrid biocomposites were produced using different stacking sequences of jute-cotton and PALF-cotton fabric plies, which were impregnated with epoxy resin. The blending fibre was chosen as cotton as it produces negligible change in the characteristics of the fabric plies [61], while allowing the pineapple fibre to be woven into a fabric [6]. Thermoset matrix, such as epoxy, is commonly used in fibre-reinforced composites as it facilitates good interaction between the matrix and fibres, thereby improving mechanical performance [62,63]. The study investigated the influence of the stacking sequence of the plies and the effects of hybridization in comparison to pure jute-cotton and pure cotton PALF epoxy-based composites. Mechanical characterization was conducted through uniaxial tensile, flexural, and impact tests. Additionally, to gain a comprehensive understanding of the fabricated biocomposites, moisture absorption and vibration properties of the hybrid NFRCs were also examined.

## 2 Materials and methods

Mercerized (alkali treated in 10% NaOH solution, at ambient temperature for 30 min, then washed in cold water and dried for 24 h) twill woven jute – cotton fabric with a surface density of 0.265 kg m^−2^ (265 GSM) and knitted PALF – cotton fabric with a surface density of 0.174 kg m^−2^ (174 GSM) was acquired from Classical Handmade BD (Dhaka. Bangladesh). The jute-cotton and PALF-cotton blend were in 55:45 and 20:80 ratios respectively. The woven jute – cotton fabric was alkali treated in order to remove the non-cellulosic substances such as hemicellulose, pectin, lignin, and wax from the fibre surface. This treatment promotes surface roughening and improves the adhesion between the fibres and the matrix. The alkali treatment is commonly performed by immersing the untreated fibres in an aqueous solution of alkali, typically sodium hydroxide (NaOH), at varying concentrations.

The jute-cotton fabric was alkali treated, whereas alkali treatment was not carried out in PALF-cotton fabric. In PALF, alkali treatment leads to complete destruction of the hemicellulose layers, and often partial destruction of lignin as well. Delignification causes reduction of tensile strength of the fibre. Hence significant improvement in mechanical properties, notably tensile strength, is not achieved at treatments in both low and high concentrations of alkali [64]. But, on the other hand, for jute fibres, the hemicellulose is degraded and lignin is preserved even at higher concentration of alkali. Significant improvement in mechanical properties such as tensile and impact strength of jute fabric were demonstrated after alkali treatment [65].

Araldite AW106 and hardener HV953 U (Huntsman Corporation, USA) epoxy resin, was used as the matrix. These epoxy components were acquired from Fixit Industries in Bangladesh and mixed at a ratio of 5:4 of resin and hardener.

### 2.1 Composite fabrication

The hardener and epoxy were mixed in a bowl using a stirrer, ensuring that air bubbles were avoided. Air bubbles can become trapped in the matrix, leading to increased void content and reduced mechanical performance of the material. To fabricate the hybrid composites, the PALF-cotton fabric ply (250 × 100 mm^2^ dimension) was placed on the die, and the resin-hardener mixture was applied by wet lay-up process. Then the jute-cotton fabric ply (also 250 × 100 mm^2^ dimension) was stacked layer – by layer prior to vacuum bagging. This process was repeated until nine (4 pineapple – cotton and 5 jute – cotton) fabric plies had been arranged in a predetermined stacking sequence.

**Fig. 2:**
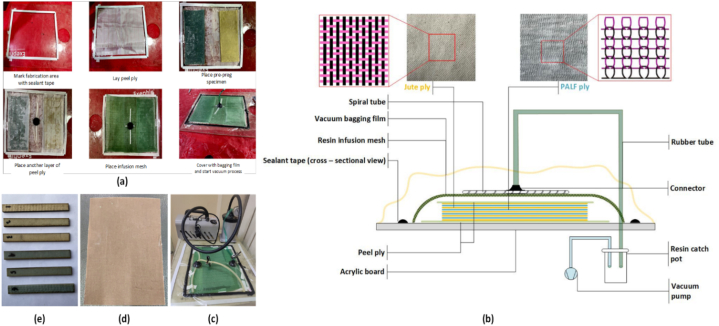
Illustration of experimental set-up for fabricating the biocomposites: **(a)** Experimental procedure for preparing specimen; **(b)** Schematic of vacuum bagging process. The acrylic board in **b** is of 10 mm thickness; **(c)** Real-life illustration of vacuum bagging process; **(d)** Fabricated specimen; **(e)** Prepared specimen for impact testing. In **e,** the dimensions are 75 mm × 10 mm.

To ensure a smooth surface finish, an epoxy coating was applied on top of the last ply before vacuum bagging. The prepreg was subjected to pressure using a vacuum pump for 1 h at room temperature. After confirming that there were no leaks in the vacuum system, the setup was left for 1 day to cure. The composites were fabricated with different hybrid ratios and stacking sequences, as listed in Table 3. The experimental set – up is illustrated in Fig. 2(a–e). Each hybrid consisted of 9 layers of reinforcement fabrics. The fibre volume fractions were found using Equation (1).

(1) $$V_{f}=\frac{\frac{W_{jute}}{Q_{jute}}+\frac{W_{PALF}}{Q_{PALF}}}{\frac{W_{jute}}{Q_{jute}}+\frac{W_{PALF}}{Q_{PALF}}+\frac{W_{R}}{Q_{R}}}$$

**Table 3:**
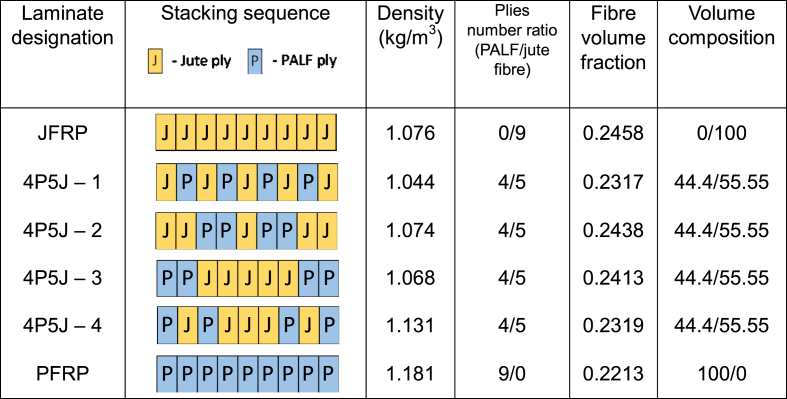
Design parameters of fabricated composites.

### 2.2 Specimen preparation

Conventional cutters, such as shear cutters, are not reliable for cutting applications due to the undesired pressure exerted from the contact edge. In contrast, laser cutting is a better alternative as: it is independent of the strength and hardness of the work material. As a consequence, it is exceptionally well-suited for cutting heterogeneous materials that consist of various phases and exhibit contrasting mechanical properties [66]. In a study conducted by Riveiro et al. [67], it was observed that the strength of synthetic fibre reinforced composites remained largely unchanged when compared to traditional mechanical machining methods.

Hence, in this experiment, CO_2_ laser cutting was employed to achieve higher precision on the cutting edges, ensuring accurate dimensions and minimizing material waste. The composites were marked and drawn according to preset ASTM standards for the subsequent tensile, flexural, impact, and moisture absorption tests. The laser cutting machine used was the Quantiumlaser Model 6040 (Set Ankara Advertising Machines, Turkey) cutting machine maintaining all the safety precautions. The laser beam was set to pulsed mode, and following the pre-marked path, it cut through the material. To achieve a finer cut, the composites in this experiment were cut using a double-pass method, as it was found to generate higher-quality cut surfaces and result in fewer burns in previous study on glass fibre reinforced polymer composites [68]. After cutting the desired pieces, any excess material was removed. As there was limited literature available on optimum cutting parameters for biocomposites, the cutting parameters in this experiment were determined through a trial-and-error approach. The chosen cutting parameters for this experiment can be found in Table 4.

**Table 4:** Cutting parameters used during CO_2_ laser cutting.

Parameters	Value
Laser intensity	90%
Speed	30 mm/s

### 2.3 Microstructure and morphology analysis

In order to analyze the fracture morphology and microstructure of the specimens before and after mechanical testing, TESCAN VEGA 3 Scanning Electron Microscope (TESCAN, Czechoslovakia) was utilized. To reveal the cross-section through the thickness, samples taken from the laminates were mounted in acrylic resin. A total of five samples were selected for examination, including one jute-cotton yarn, one pineapple-cotton yarn, a pineapple-cotton fibre reinforced epoxy polymer composite (PFRP), a jute-cotton fibre reinforced epoxy polymer composite (JFRP), and a cotton blended pineapple-jute fibre reinforced hybrid composite. The yarns used in the experiment were derived from the fabrics employed, while the composite samples were obtained by CO_2_ laser cutting from the fractured portions of the tensile testing specimens, with a dimension of 10 mm. These samples were then mounted onto a sample holder. Prior to SEM imaging, the specimens were sputter-coated with gold dust for 1 min. This coating helps to improve the conductivity of the sample and minimize charging effects during imaging, enabling better quality SEM images to be obtained.

### 2.4 Mechanical characterization

#### 2.4.1 Tensile test

The rectangular – shaped specimen were subjected to uniaxial tensile tests using the Shimadzu AGX – V2 (Shimadzu Corporation, Tokyo, Japan) testing machine. The standard head displacement of 2 mm/min was applied during the test. The dimensions of the specimen were taken to be 250 × 25 mm^2^ in accordance to ASTM D3039/D3039M − 17 [69].

#### 2.4.2 Flexural test

In this experiment, the flexural properties of the composites were determined using a three-point bending test conducted on the Shimadzu AGX-V2 series (Shimadzu Corporation, Tokyo, Japan) testing machine. The procedure involved placing a bar-shaped composite specimen on two supports, with a loading nose applied at the midpoint between the supports. The flexural test was performed according to the guidelines provided in ASTM standard D6856 [70]. The span-to-thickness ratio was set at 25:1, with the standard specimen thickness averaging 4 mm and the standard width averaging 13 mm. The length of the specimen was 100 mm. During the test, the load was applied at the midpoint of the specimen at a cross-head speed of 5 mm/min.

The flexural stresses of the composites were calculated using Equation (2):

(2) $$\sigma =\frac{3PL}{2bh^{2}}$$

where,

σ = stress at the outer surface at mid-span, MPa;
*P* = applied force, N;
*L* = support span, mm;
*b* = width of beam, mm;
*h* = thickness of beam, mm.

The flexural modulus is calculated using the following Equation (3):

(3) $$E_{f}=\frac{\Delta \sigma }{\Delta \varepsilon }$$

where.

$E_{f}$ = flexural modulus of elasticity, MPa;
Δσ = difference in flexural stress between the two selected strain points, MPa;
Δε = difference between the two selected strain points (nominally 0.002).

The specific modulus refers to a characteristic of a material that indicates the ratio of stiffness to weight. It is a measure of the material's ability to resist deformation under an applied load, relative to its weight. Specifically, it is calculated as the ratio of the material's modulus of elasticity (which is also known as Young's modulus) to its density.

The specific modulus is calculated by the following Equation (4):

(4) $$SM=\frac{E_{f}}{\rho }$$

where,

*SM* = Specific modulus of elasticity;
$E_{f}$ = flexural modulus of elasticity, MPa;
*ρ* = density of the composite, kg/m^3^.

These equations provide the necessary formulas to calculate these properties based on the test data obtained from the three-point bending test.

#### 2.4.3 Impact testing

According to the International Standard ISO:1997 Standard [71], unnotched samples were prepared by CO_2_ laser cutting, and a modified Charpy test was carried out on an impact testing machine, specifically the Instron CEAST 9050 (Instron Corp., Norwood, MA, USA). The dimensions of each sample were taken 75 × 10 × 4 mm^3^ with a span-to-depth ratio set at 18. This ratio indicates the distance between the supports of the sample relative to its depth. During the test, the sample was placed in the testing machine and was positioned horizontally, and supported at both ends. The pendulum of the machine was released with a velocity of 5.35 m/s and swung down to strike the sample. By conducting the modified Charpy test, the impact properties of the composites, such as impact strength and energy absorption, could be determined. The test allowed for the evaluation of the material's resistance to sudden applied loads and its ability to absorb energy without fracture.

#### 2.4.4 Moisture absorption testing

For the moisture absorption test, the sample dimensions were in accordance with ASTM D5229/D5229M − 20 [72] with a size of 50 × 50 mm^2^. The samples were immersed in a body of water at ambient temperature, and the mass of each sample was taken for 21 days.

Equation (5) was used to calculate the moisture absorption in percentage:

(5) $$\text{Moisture}\;\text{absorption}\;\left(\%\right)=\frac{m_{2}-m_{1}}{m_{1}}\times 100$$

where m_2_ is the weight after immersion, and m_1_ is the oven – dry mass.

#### 2.4.5 Vibration testing

**Fig. 3:**
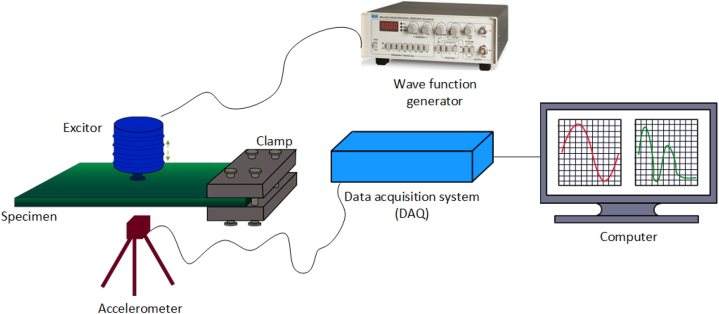
Schematic of the test set up to find the first mode natural frequency of the composite specimens.

The dynamic properties of the samples were examined by exciting composite samples with an exciter and examining the frequency responses. The magnitude versus frequency responses were calculated using the Fast Fourier Transform (FFT) within a fixed frequency range of 10–120 Hz. Six specimens of 250 × 100 mm^2^ were created for each laminate arrangement, and vibration tests were conducted, with each specimen fixed on one side (cantilever condition). A schematic of the test set up is shown in Fig. 3.

**Fig. 4:**
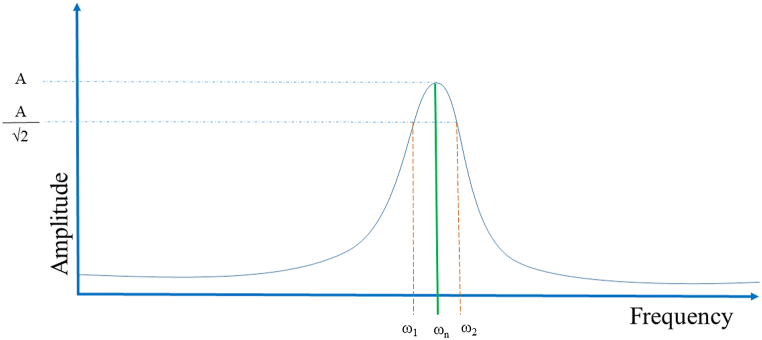
Half bandwidth method (ω_n_ is the natural frequency at first mode, ω_1_, ω_2_ is the frequency at 1/ $\sqrt{2}$ of the maximum amplitude).

The damping ratio of the specimens were calculated by the ‘half-bandwidth method’ [73,74] as shown in Fig. 4 using Equation (6)

(6) $$\zeta =\frac{\omega_{2}-\omega_{1}}{2\omega_{n}}$$

#### 2.4.6 Statistical analysis

One-way ANOVA test was conducted using Minitab 20 for a total of 5 samples for the mechanical tests. The confidence interval had been kept at 95%, meaning that the p-value less than 0.05 would result in the rejection of the null hypothesis.

## 3 Results and discussion

### 3.1 Microstructure of jute – cotton and PALF – cotton yarns

The microstructures of pineapple-cotton reinforced polymer composites, jute-cotton reinforced polymer composites, and hybrid composites were observed using scanning electron microscopy (SEM). Images were captured after tensile fracture to analyze the causes of failure and strength reduction. Additionally, the microstructures of both pineapple-cotton and jute-cotton yarns were also examined.

**Fig. 5:**
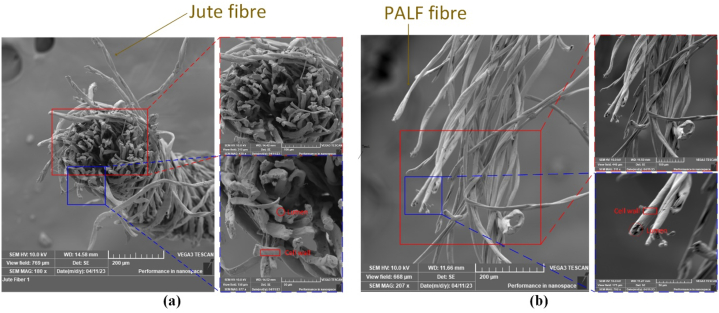
**(a)** Scanning electron micrographs of jute fibre yarn in different magnifications and positions at 200 μm magnification; 100 μm magnification; 50 μm magnification; **(b)** Scanning electron micrographs of PALF yarn in different magnifications and positions at 200 μm magnification; 100 μm magnification; 50 μm magnification; In the bottom right panel of **(a)** and **(b),** the dark spots are the lumen and the surface of the fibres is the cell wall.

The varying magnifications of the images provide a clearer understanding of the materials' structure. Fig. 5(a) shows the jute-cotton yarn, and at magnifications of 200 μm and 100 μm, the fibre orientation becomes evident. At a magnification of 50 μm, it is possible to distinguish the lumen and cell wall of the jute fibres to some extent. Fig. 5(b) depicts the fibres in the pineapple-cotton yarn, revealing their cross-section at a magnification of 50 μm. The cross-sectional geometry of jute fibres is shown in Fig. 5(a) using SEM images, which reveals an uneven form with different diameters along the fibre's length [75]. The fibre's surface shape appears rough and is characterized by sporadic micropores (lumen). On their surfaces, immature fibres often have fewer micropores [76]. The SEM images reveal a more aligned structure for the jute-cotton yarn when compared to the yarn made by PALF-cotton. The greater fibre alignment indicates greater anisotropic yarn strength. In the subsequent sections, it will be made clear how the overall architecture and arrangement of fibres can influence the composite properties.

### 3.2 Tensile properties of the composites

**Fig. 6:**
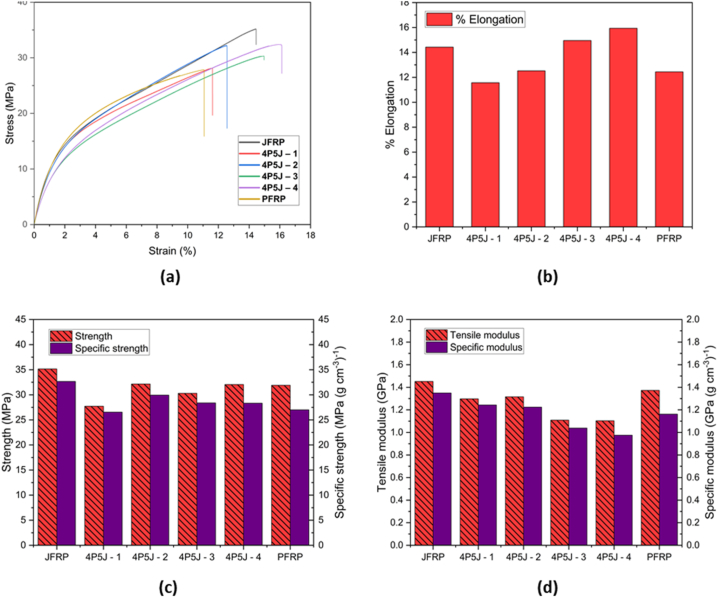
**(a)** Tensile stress - strain curve of fabricated composites; **(b)** % Elongation of fabricated composites; **(c)** Strength (left bar) and specific tensile strength (right bar) of fabricated composites; **(d)** Tensile modulus (left bar) and specific tensile modulus (right bar).

The typical tensile properties of both the pure jute-cotton, hybrid and pure PALF-cotton composite along the longitudinal direction are shown in Fig. 6(a–d). Increasing the volume fraction of jute resulted in a higher tensile modulus and strength. For JFRP, the values were observed to be 1.45 GPa and 35.16 MPa, respectively, while for PFRP, the values were 1.37 GPa and 31.91 MPa, respectively. It was expected that neat JFRP should have higher modulus and strength compared to PFRP as the mechanical performance of jute is stronger than PALF. These findings support the expected strengthening and stiffening effect of incorporating jute into the hybrid. However, PFRP exhibited greater ductility and did not show a definitive yield point.

The highest breaking stress was observed for JFRP (35.16 MPa), while the lowest was for 4P5J – 1 (27.73 MPa), indicating a decrease in tensile strength by 26.67% and only a 5.84% decrease in tensile modulus. Fig. 6(c–d) illustrates that the strength and modulus varied depending on the stacking sequence, with the highest values found for 4P5J – 2 (32.16 MPa). This variation could be attributed to poor interfacial adhesion between the jute-cotton and PALF-cotton plies, which is more pronounced in 4P5J – 1, resulting in its lower breaking stress. The number of jute/PALF interfaces are 8,4,2 and 6 for 4P5J-1, 4P5J-2, 4P5J-3 and 4P5J-4 respectively. Higher number of jute/PALF interfacial layers, especially when the interface is poor can create inadequate stress transfer and promote matrix cracking, negatively effecting the strength of the fibre reinforced hybrids. Putting plies of the same type of material together in the composite generally led to an increase in tensile strength as it reduces the number of jute/PALF interfaces and improves interfacial adhesion. This is predicted to be the reason why 4P5J-1 showed the lowest tensile strength, despite having similar volume fraction of PALF/jute fibres of other specimens. For 4P5J-2 and 4P5J-4, there is no significant difference in the tensile strength. However, 4P5J-3 had the second lowest tensile strength, despite having the lowest number of PALF/jute interfaces. This can be attributed to the outer layers being made from PFRP, whose brittle nature likely initiated first ply failure, which propagated to the next PALF ply, ultimately resulting in early failure [77]. Hence, the stacking sequence has a significant impact on the tensile strength of hybrid NFRCs.

As per classical laminate theory, it is expected that tensile modulus of specimens should be similar given that they have similar volume fraction of fibres and contain same numbers of jute and PALF plies. In our case, the variation of tensile modulus can vary due to interlayer bond strength. Higher values of tensile modulus are likely to be observed for alternate layers of plies for hybrid composite [78]. Likewise, in this study, the relatively high tensile modulus of 4P5J-1 (1.243 GPa), with alternate layers of PALF and jute emulates that result. The tensile modulus was increased significantly (by 19.2%) when the jute-cotton fibre plies were concentrated on the outer edges, rising from 1.103 GPa (4P5J – 4) to the highest of 1.315 GPa (4P5J – 2). This may be due to the higher stiffness of the jute fibre, which lets the hybrids take in more energy before striations were observed, which led to delayed formation of stress concentration regions.

A positive hybrid effect is shown in 4P5J-3 and 4P5J-4 in terms of elongation. PFRP exhibited less elongation (12.44%) compared to JFRP (14.43%). However, the hybrid composite demonstrated the highest elongation in 4P5J – 4 (15.94%). Interestingly, although the fibre volume fraction of jute and PALF are not significantly different from the hybrids, the stacking sequence played a major role on the elongation till break behaviour of the composites. In terms of elongation, jute plies introduced a synergistic effect when it is incorporated with PALF plies into the matrix material. It is predicted that, the ductile stacked jute layers in the mid region of 4P5J-3 and 4P5J-4 carried the stress after brittle fracture of outer PALF layers, hence improving the elongation to break.

Since the density of jute is higher than that of PALF, the hybrid composites exhibited slight improvements in specific strength and moduli when jute was incorporated into the pure PALF-cotton composite. Notably, 4P5J – 2 demonstrated the highest specific strength of 29.94 MPa (g cm^−3^)^−1^, while 4P5J – 1 exhibited the highest specific modulus of 1.24 GPa (g cm^−3^)^−1^.

**Fig. 7:**
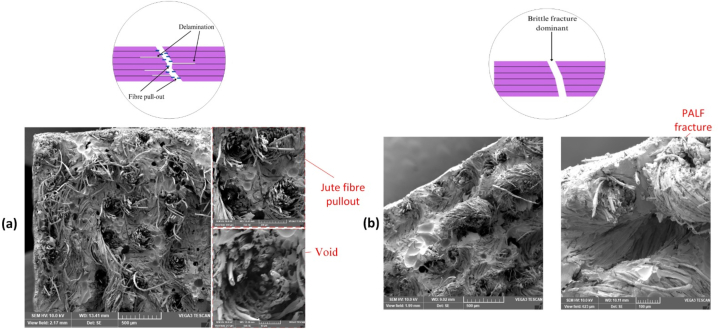
SEM image and schematic of tensile fracture of **(a)** JFRP composite at 500 μm (left panel), 200 μm (top right panel) and 50 μm (bottom right panel), and **(b)** PFRP composite at 500 μm (left panel) and 100 μm (right panel).

Visual inspection, as shown in Fig. S1 (see in supplementary text), revealed that, during tensile failure, fibre yarn pull-out was more pronounced in JFRP, while PFRP exhibited a nearly brittle fracture. SEM images in Fig. 7(a–b) demonstrate the dispersion and distribution of fibres within the composite materials for JFRP and PFRP, respectively. The presence of voids in the images indicate a reduction in composite strength. Despite JFRP showing maximum tensile performance, it was evident that extensive fibre pull-out on the tension side of the fracture, indicating poor fibre-matrix adhesion, likely reduced the composite strength, even after alkali treatment. Additionally, the images show fibre agglomeration, where fibres stack collectively in the matrix, and non-uniform stress transfer, both contributing to reduced strength. Therefore, factors such as fibre-matrix adhesion, fibre dispersion and orientation, fibre agglomeration, and presence of voids all play influential roles in the reduction of strength of the fibre-reinforced biocomposites.

**Fig. 8:**
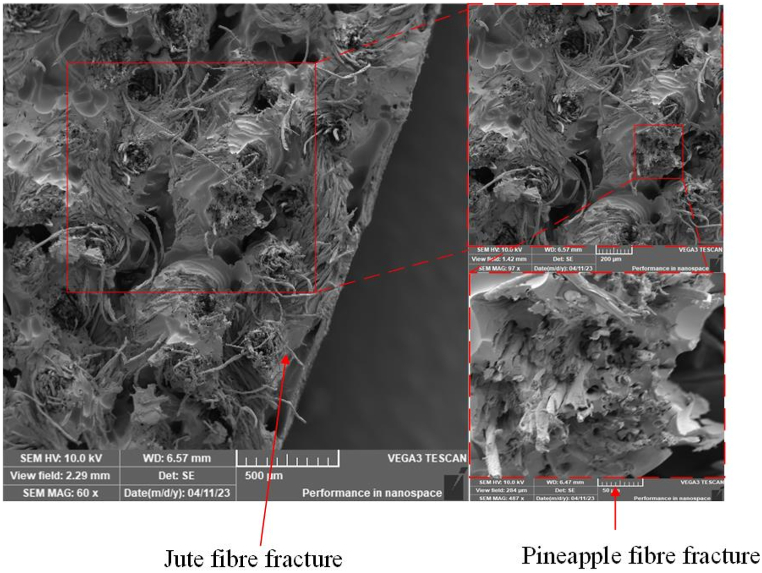
SEM image of tensile fracture of hybrid composite 4P5J - 1 at 500 μm (left panel), 200 μm (top right panel) and 50 μm (bottom right panel).

SEM images of the fabricated hybrid composites are presented in Fig. 8. In comparison to the smooth fracture observed in PFRP, indicating a dominant brittle fracture, the hybrid composite 4P5J – 1 exhibits a more intricate fracture pattern. This pattern includes jute fibre pull-out, jute-cotton yarn pull-out, and ductile fracture of jute-cotton yarn. These distinctive features are more prominently observed in the fracture morphology of JFRP, as shown in Fig. 7(a). As alkali treatment is particularly well-suited for enhancing the tensile strength of jute fibres compared to other treatments tested, the chemical treatment of the jute fibre is expected to deliver greater performance [79]. Thus, the untreated pineapple fibre and the stacking sequence are possibly responsible for the relatively reduced tensile strength in the hybrid composites.

Overall, the hybridization of jute and pineapple to fabricate a composite resulted in a significant improvement in elongation, albeit with a slight decrease in tensile strength and modulus compared to JFRP.

### 3.3 Flexural properties of the composites

**Fig. 9:**
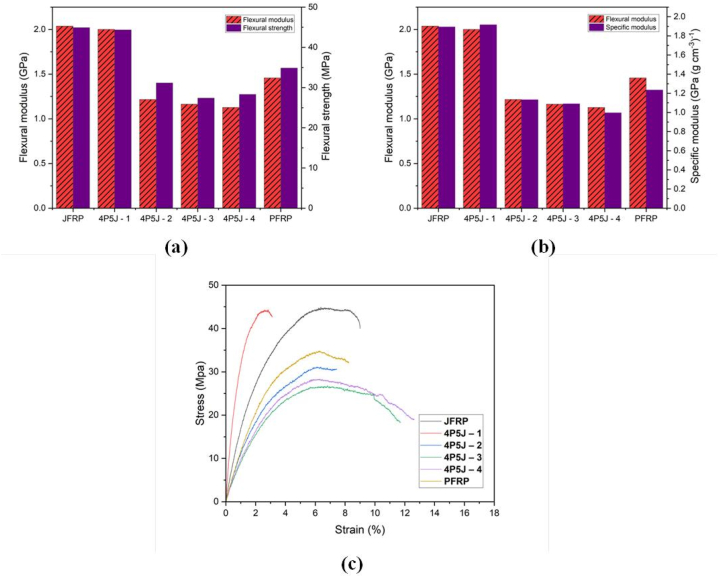
**(a)** Flexural modulus (left) and flexural strength (right) of fabricated composites; **(b)** Flexural modulus (left) and specific flexural modulus (right) of fabricated composites and **(c)** Typical flexural stress–strain curves of fabrication composites.

In general, structural materials with high tensile strength tend to perform well when subjected to bending loads due to their favorable interaction between the fibres and the matrix. The study investigated the flexural properties of the materials, as presented in Fig. 9(a–c). Unlike the tensile behaviour, the flexural stress-strain curve shown in Fig. 9(c) exhibited significant variations in flexural stress, with the majority of composites demonstrating a continuous, one-step failure.

4P5J – 1, the first hybrid composite, exhibited higher strength (44.36 MPa) compared to both PFRP (33.85 MPa) and the other hybrid composites. It is found that composite specimens that have alternate layers of jute and pineapple leaf fibre (4P5J-1) has shown the highest flexural modulus and strength amongst the hybrid composites. It is likely, that less interlayer delamination is caused due to the reduction of lamina thickness by stacking dissimilar materials stacked on top of another. This effectively slows down the crack propagation hence, higher flexural modulus and strength is achieved. Supporting arguments were noticed in studies on flexure modulus of hybrid NFRCs [78,80].

A decreasing trend in strength is observed from JFRP to 4P5J – 3, followed by an inverse trend from 4P5J – 4 to PFRP, as illustrated in Fig. 9(a). A similar trend was observed for the flexural modulus, except for the PFRP composite. Notably, the first hybrid composite, 4P5J-1, exhibited a significant improvement in flexural modulus, increasing by 78.57% (from 1.12 GPa in 4P5J - 4 to 2.00 GPa in 4P5J-1), with the only variation being the stacking sequence. The flexural strength also increased by approximately 56.80%, from the lowest value of 28.29 MPa in 4P5J - 4 to 44.36 MPa in 4P5J-1. This improvement may be attributed to the lower elongation of the hybrid composite, enabling it to absorb more energy before failure.

However, it is important to note that JFRP composite exhibited the highest values in both flexural modulus and strength, with respective values of 2.03 GPa and 44.91 MPa. Although all of the composites exhibited flexural strength values that were either higher or comparable to those of PFRP, the increase in flexural strength was not as significant as the increase in tensile strength. The relatively poor flexural strength observed in the hybrid composites could be attributed to the relatively weaker fibre strength and/or insufficient interfacial adhesion [81].

By comparing the results of the modulus and strength in the hybrid composites, it can be concluded that optimizing the stacking sequence could lead to improved flexural performance. Considering this concept, hybridization of the plies in a 1:1 stacking sequence could be employed to withstand both the highest normal stress on the outside and the highest shear stress on the inside, particularly under three-point bending.

Based on Fig. 9(b), it can be observed that the specific modulus of the composites follows a similar trend to the flexural modulus, except for PFRP, which exhibits a higher flexural modulus than specific modulus. The highest specific modulus is observed in 4P5J – 1 (1.91 GPa (g cm^−3^)^−1^), which surpasses JFRP (1.89 GPa (g cm^−3^)^−1^), while 4P5J - 4 exhibits the lowest value of 0.99 GPa (g cm^−3^)^−1^. Thus, it can be concluded that the first sample of the hybrid composite, 4P5J – 1, is the most rigid for its weight among the other composites. This observation further emphasizes the significant role of the stacking sequence in achieving optimal flexural performance for the composites.

### 3.4 Impact properties of the composites

**Fig. 10:**
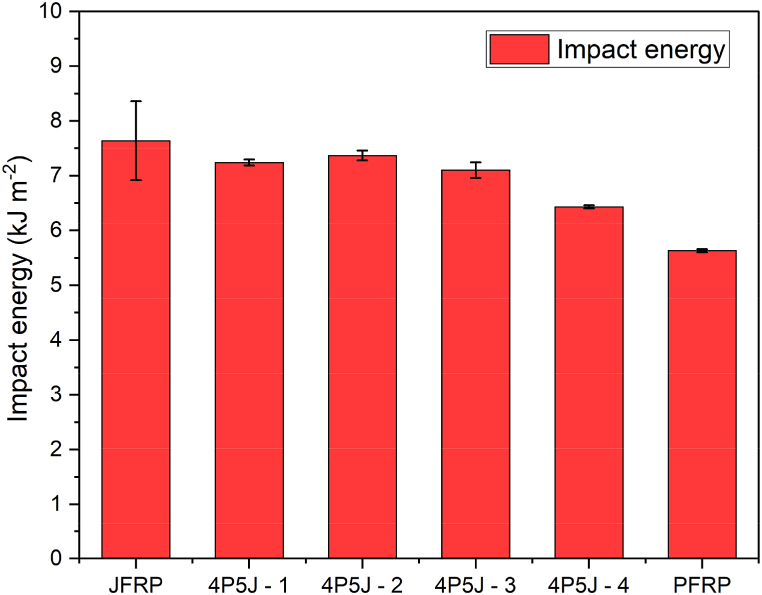
Impact energy values of the fabricated composites.

The impact energy values shown in Fig. 10 represent the impact strengths of the composites. The highest impact energy was observed in JFRP. However, a decreasing trend can be observed among the other composites except for 4P5J-2, which exhibited an impact energy (7.236 kJ/m^2^) close to that of JFRP (7.638 kJ/m^2^) and displayed pull-out behaviour. This behaviour can negatively affect the impact behaviour of the NFRC [82]. On the other hand, PFRP exhibited the lowest impact energy value of 5.628 kJ/m^2^. When compared to JFRP, which fractured at impact energies greater than 7 kJ/m^2^, the hybrid composites and PFRP demonstrated less plastic deformation in a prolonged fracture process at impact energies below 7 kJ/m^2^.

Specimens that had jute plies at the surface as a ‘skin’ showed higher impact energy compared to those specimens having PALF the outermost plies and as skin. The outermost layer of jute has more strength and is capable of absorbing more impact energy than PALF. Furthermore, 4P5J-2 showed highest impact energy absorption, possibly due to having more outermost jute plies. For NFRC hybrids, layering the outermost regions with high strength fibre plies have been showed to be effective in increasing the impact energy absorption [83]. Hence, the stacking sequence had a noticeable effect on the impact energy absorption of the composites.

### 3.5 Other properties of the fabricated composites

#### 3.5.1 Moisture absorption properties

The hydrophilic nature of natural fibres can greatly influence the performance of a composite reinforced with them [54]. Factors such as the type of plastic, additives used, temperature, and duration of exposure can all impact moisture absorption [84].

**Fig. 11:**
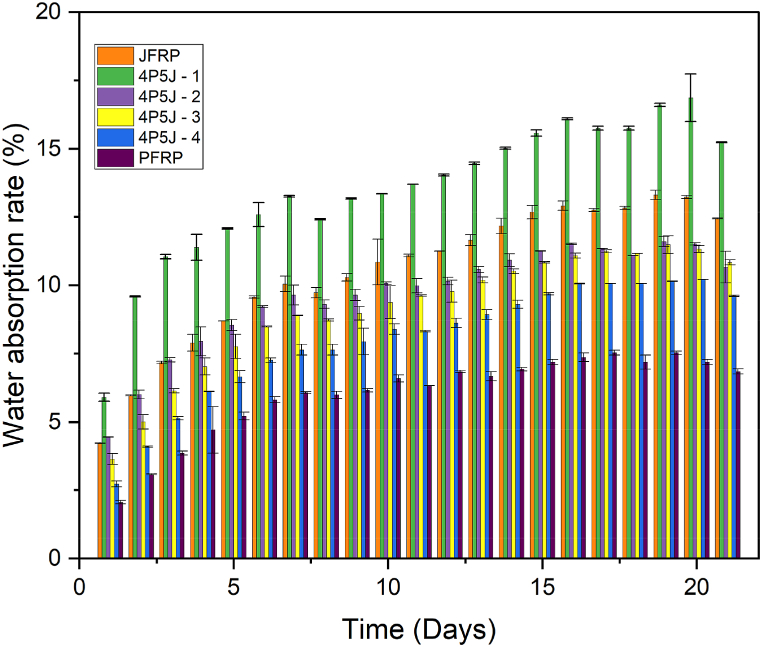
Moisture absorption (in %) w. r.t time (in days); please refer to the digital copy of the article to see the different colors labelling the different composite specimens.

The moisture absorption test revealed that all the composites exhibited an overall increase in mass over time, as shown in Fig. 11. It was observed that all the composites showed a general increase in mass with each day. In about 6 days, the moisture absorption of hybrid composites and PFRP approached a plateau where the rate of absorption decreased but did not become constant. Furthermore, there was a dip in moisture absorption at day 8 and day 18 before reaching a maximum point in day 20.

After that, moisture desorption was observed. In the case of JFRP, we can see that the initial rate of moisture absorption is significantly high, reaching its plateau in about 4 days. It was observed that, for 4P5J – 1, the hybridization of pineapple increased the moisture absorption significantly (an increase of 22.5% compared to JFRP) after 21 days. This could be attributed to a combination of factors, including the presence of more hydroxyl groups in the jute-cotton fibre ply (which can form hydrogen bonds with water molecules, leading to water retention [85]), which was on the outside of the composite, and poor interfacial adhesion between the alternating jute/PALF plies and the matrix, facilitating enhanced moisture absorption through capillary effect and microcracking [86]. The presence of voids observed in the SEM micrograph of 4P5J-1 may also contribute to water retention.

On the other hand, for all the other composites, hybridization effectively reduced moisture absorption to approximately 16.70%, 14.84%, and 29.50% for 4P5J – 2, 4P5J – 3, and 4P5J – 4, respectively, when compared to the moisture absorption of 12.44 wt% JFRP after 21 days. The significant reduction in moisture absorption may be attributed to a lower presence of hydroxyl groups on the composite surface [85] as well as improved surface adhesion when stacking the same type of plies together.

In conclusion, hybridization with pineapple leaf fibres appears to mitigate the moisture absorption issue in jute fibre-reinforced composites, with the exception of 4P5J – 1, where the hybridization increased moisture absorption. This suggests that careful selection of hybridization sequence and optimizing the interface between fibre plies and the matrix can help alleviate moisture absorption problems in these composites.

#### 3.5.2 Vibration properties

Hybridization has been reported to be an effective method in improving the damping performance of natural fibre reinforced composites [87]. Therefore, the stacking sequence will have a significant effect on the vibration and damping properties of a laminated NFRCs. Generally, NFRCs with high stiffness are expected to give higher natural frequencies and lower damping properties [82,88]**.**

**Fig. 12:**
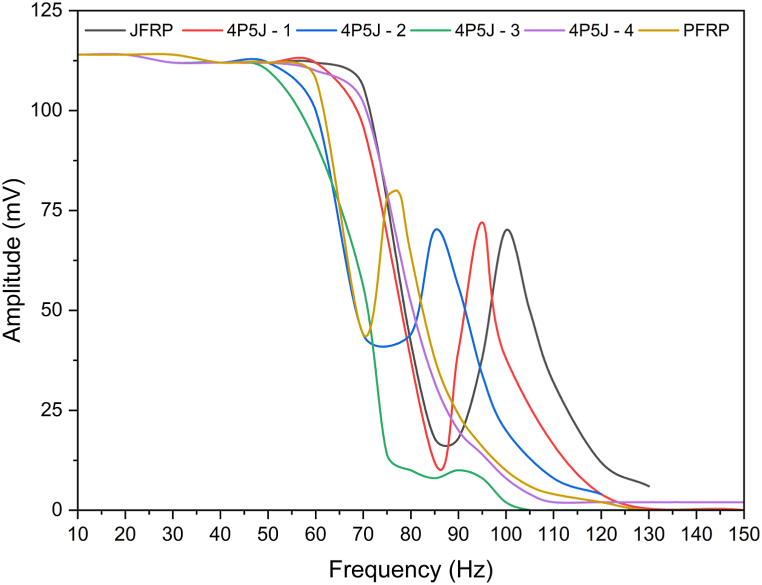
Frequency response curves of fabricated composites.

The frequency response curves are presented in Fig. 12, revealing that all samples primarily exhibit the first mode natural frequency.

At a frequency of 10 Hz, all composite samples displayed an identical amplitude of 114 mV. However, within the frequency range of 50 Hz–70 Hz, different composite laminates produced noise of varying amplitudes. The 4P5J-3 and 4P5J-4 specimens showed negligible amplitude at a frequency of 120 Hz, while the remaining samples displayed similar behaviour between 120 Hz and 140 Hz. This observation can be attributed to limitations in the vibration testing system. It is possible that the exciter used in the tests exceeded its maximum capacity or limit. Once this threshold is reached, the vibration amplitude no longer increases, resulting in a flattened response.

In Table 5, the recorded natural frequency for first mode of JFRP and PFRP are 102 Hz and 76 Hz respectively, and the damping ratio of JFRP and PFRP are 0.044118 and 0.085526 respectively. JFRP showed highest first mode natural frequency and lowest damping ratio among the specimens. The damping-natural frequency trend of the tested specimens in our study is that, the higher the first mode natural frequency, the lower the damping ratio. Such trend has been reported in hybrid NFRCs in literature as well [82]. Besides, the specimens that have higher number of stiff fibres on the outer plies shows higher damping ratio, and lower natural frequency [89]. This may be due to the stiffer fabric on the outer surface conveyed the vibration energy to the less stiff fibres in the core, thus dissipating energy internally [90].

**Table 5:** First mode natural frequency of tested specimens.

Sample Name	First mode natural frequency in Hz	Damping Ratio
JFRP	102	0.044118
4P5J- 1	95	0.057895
4P5J- 2	85	0.070588
PFRP	76	0.085526
*4P5J-3 and 4P5J-4 are not shown as they did not show any first mode natural frequencies		

However, notable spikes in amplitude were not observed when the PALF-cotton ply was stacked on the outside of the laminated composite (as depicted in Fig. 12 for 4P5J-3 and 4P5J-4). This phenomenon may be attributed to the system becoming critically damped as the amplitude v/s frequency curve (Fig. 12) depicts. In any case, these two specimens are better in damping performance. This could be attributed to the enhanced vibration absorption provided by the knitted weave pattern of the PALF-cotton ply, which features a spiral orientation of yarns and relatively larger gaps between them [91]. Another contributing factor could be the different weave patterns of the jute-cotton and PALF-cotton plies, which can hinder energy transfer due to variations in the patterns of gaps between the yarns. PFRP and JFRP exhibited an intermediate peak due to sufficient contact between the plies, as they all shared the same weaving pattern, facilitating easier energy transfer.

These findings highlight the significance of considering resonance phenomena and system limitations when conducting vibration testing.

### 3.6 Statistical analysis results

The mean and standard deviation of the tensile strength, flexural strength and impact strength of the fabricated composites are given in Table 6.

**Table 6:** Mean and standard deviation of fabricated biocomposites.

Sample name	Mean of tensile strength in MPa	Standard deviation of tensile strength in MPa	Mean of flexural strength in MPa	Standard deviation of flexural strength in MPa	Mean of impact strength in kJ/m^2^	Standard deviation of impact strength in kJ/m^2^
JFRP	35.16	0.377	44.91	1.569	7.64	0.403
4P5J-1	27.73	0.279	44.36	1.619	7.24	0.0344
4P5J-2	32.16	0.289	31.14	0.583	7.37	0.0541
4P5J-3	30.30	0.052	27.37	0.249	7.10	0.0894
4P5J-4	32.05	0.239	28.29	0.211	6.43	0.0152
PFRP	31.91	1.683	33.85	0.795	5.63	0.403

**Fig. 13:**
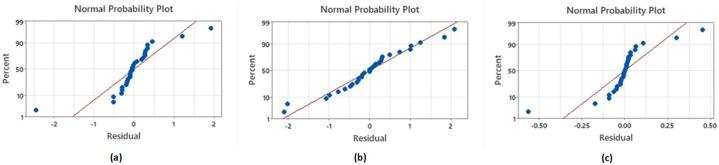
Normal probability plots for **(a)** Tensile strength, **(b)** Flexural strength and **(c)** Impact strength.

In Table 7, the degree of freedom, adjacent sum of the square, adjacent mean of square, F-value and *p*-value has been reported. It can be observed from Table 7 that the p-values are less than 0.05, indicating a statistically significant difference of the mechanical properties of the hybrid composites. The normal probability plots shown in Fig. 13(a–c) shows that the values are approximately normally distributed.

**Table 7:** One-way ANOVA for **(a)** Tensile strength, **(b)** Young's modulus and **(c)** Impact strength.

Source	Degree of freedom	Adjacent sum of square	Adjacent mean of square	F-Value	p-value
**(a) Tensile Strength**					
Between the group	5	136.53	27.3062	51.26	0.000
Within the group	24	12.78	0.5327	–	–
**(b) Flexural Strength**					
Between the group	5	1239.54	247.908	241.33	0.000
Within the group	24	24.65	1.027	–	–
**(c) Impact Strength**					
Between the group	5	14.181	2.83682	97.37	0.000
Within the group	24	0.6992	0.02913	–	–

### 3.7 Potential applications of the fabricated hybrid biocomposites

**Fig. 14:**
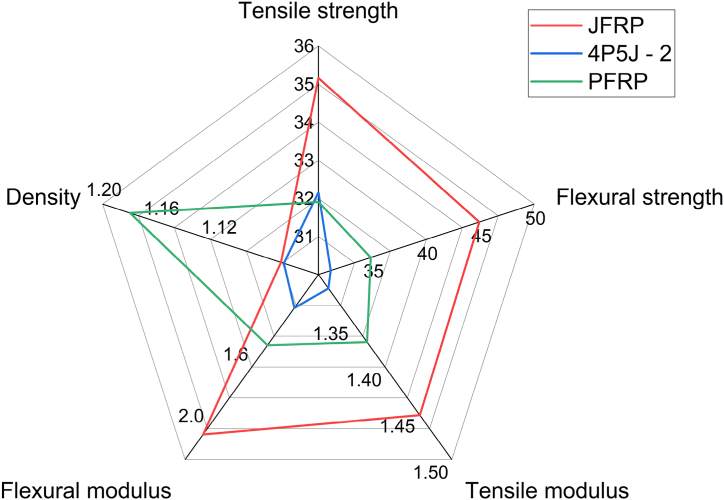
Comparative radar plots of key mechanical properties: Tensile strength (MPa); Flexural strength (MPa); Tensile modulus (GPa); Flexural modulus (GPa); Density (kg m^−3^).

The comparative plot presented in Fig. 14 illustrates the tensile and flexural strength, tensile and flexural modulus, and density of JFRP, PFRP, and 4P5J-2. It is evident that JFRP exhibits the highest mechanical properties in all parameters, except for density, where PFRP demonstrates dominance. The hybrid composite, 4P5J-2, falls between JFRP and PFRP in terms of mechanical properties, with the exception of density, which is slightly lower than that of JFRP. These specific mechanical properties of the fabricated hybrid composite can be used for automotive applications such as brake, accelerator, clutch pedals, as well as mounting brackets [92,93]. Mechanical properties such as tensile strength and flexural strength obtained from hybridization in our study are suitable for NFRC application in automotive structural parts as well [94]. Furthermore, due to its low density and cost (around $100 per composite), further enhancements in the other parameters displayed in Fig. 14 could enable its utilization in other automotive applications such as dashboards and bonnets, as well as non-strength critical aerospace applications like airplane interiors. In the future, to maintain environmental sustainability, surface treatments can be applied to the reinforcements to improve interfacial strength, which was assumed to be the primary limitation in our experiment. Hybridization with stronger fabrics like kenaf, ramie, and flax can also be explored, opening up possibilities for bioengineering applications such as bone tissue scaffolding, provided a bio-based matrix is utilized.

## 4 Conclusion

This paper introduces four different stacking sequences of a hybrid biocomposite made of epoxy matrix reinforced with waste cotton-blended jute and pineapple leaf fibres (PALF) fabrics from the textile industry. Overall, all the hybrid composites exhibited improved mechanical properties compared to the pineapple leaf fibre reinforced polymer (PFRP) composite, but fell short of the strength of the jute fibre reinforced polymer (JFRP) composite. SEM micrography of the fibres demonstrated that the jute yarn had more aligned fibres than PALF yarns, which leads to jute-cotton fabric's greater anisotropic strength. SEM images of the composites after tensile fracture also revealed that JFRP showed a ductile fracture with yarn pull-out, whereas PFRP showed a much more brittle fracture. It is predicted that, due to poor adhesion, the hybrids with more jute/PALF interfacial layers exhibited decreased tensile strength, as observed in the hybrid made of alternating jute/PALF plies (4P5J-1). Furthermore, concentrating PALF plies on the outer regions of the composite can lead to early failure, regardless the number of interfacial layers, as the brittle nature of PALF can cause quick first ply failure, leading to stress concentration regions (from 4P5J-3). Hybrid composite 4P5J-4 demonstrated a balance between these two constraints and hence, showed a significant increase in elongation (15.94%), and the highest tensile strength (32.16 MPa) among the hybrid composites. However, due to the reduction in lamina thickness by stacking dissimilar materials together, alternating jute/PALF plies to fabricate the composite displayed the highest flexural strength, as shown by 4P5J-1 (44.36 MPa), which was comparable to JFRP (44.91 MPa) under 3-point bend conditions. In terms of impact strength, hybridization increased the impact energy, to a maximum approximately 28.59% in 4P5J-2 (7.236 kJ/m^2)^ compared to PFRP (5.628 kJ/m^2^). Improved impact performance of the composites can be attributed to stacking high fibre strength plies in the outside, as well as a complex fracture pattern, as indicated by the SEM fractography of the multi-fracture surface. Moisture absorption tests demonstrated that hybridization can reduce the hydroxyl groups in the composite which, coupled with a relatively stronger interfacial adhesion, can lead to the highest reduction in moisture absorption, as observed in 4P5J-4 (29.59% decrease compared to JFRP, resulting in 9.61% moisture absorption). Due to the different weave patterns of the reinforcing materials, vibration tests revealed that hybridization generally improved the damping ratio. Furthermore, putting PALF plies on the outer layers critically damped the composite specimen. Although further studies on the mechanics of complex geometry are required, this study confirmed that the performance of the hybrid composite material consisting of PALF and jute reinforcement can be utilized for manufacturing brake, accelerator, clutch pedals as well as mounting brackets in automobiles. This study provides evidence that hybridization of natural fibres can offer potential solutions for producing composite materials tailored to specific application requirements, particularly when utilizing waste materials as reinforcements. It also expands the potential application of PALF as a reinforcement material.

## Author contribution statement

Tajwar A. Baigh; Fairooz Nanzeeba: Performed the experiments; Analyzed and interpreted the data; Wrote the paper. Hasibur R. Hamim: Performed the experiments; Wrote the paper. Mohammad Ahsan Habib: Conceived and designed the experiments; Performed the experiments; Contributed reagents, materials, analysis tools or data.

## Data availability statement

Data will be made available on request.

## Funding sources

This research did not receive any specific grant from funding agencies in the public, commercial, or not-for-profit sectors.

## Declaration of generative AI and AI-assisted technologies include the writing process

During the preparation in this employment the author(s) used ChatGPT in order to proofread and improve the clarity and coherency of the writing. After employing this tool/service, the author(s) reviewed the edited the content as needed and take(s) complete responsibility for the content of the public.

## Declaration of competing interest

The authors declare that they have no known competing financial interests or personal relationships that could have appeared to influence the work reported in this paper.
