# Supplementary material for: A comprehensive study on the effect of hybridization and stacking sequence in fabricating cotton-blended jute and pineapple leaf fibre biocomposites
Source: Heliyon. 2023 Sep 7;9(9):e19792. doi: 10.1016/j.heliyon.2023.e19792 (PMC10559121; doi:10.1016/j.heliyon.2023.e19792)
Supplement: Multimedia component 1 [file mmc1.docx]

**Supplementary Information**

**A Comprehensive Study on the Effect of Hybridization and Stacking Sequence in Fabricating Cotton-blended Jute and Pineapple Leaf Fibre Biocomposites**

Tajwar A. Baigh ^a,1^; Fairooz Nanzeeba ^a,1^; Hasibur R. Hamim ^a^, M. Ahsan Habib ^a, *^

^1^Both authors contributed equally.

^*^Corresponding Author, Email: [mahabib@iut-dhaka.edu](mailto:mahabib@iut-dhaka.edu)

^a^ Department of Mechanical and Production Engineering, Islamic University of Technology (IUT),

Gazipur 1704, Bangladesh


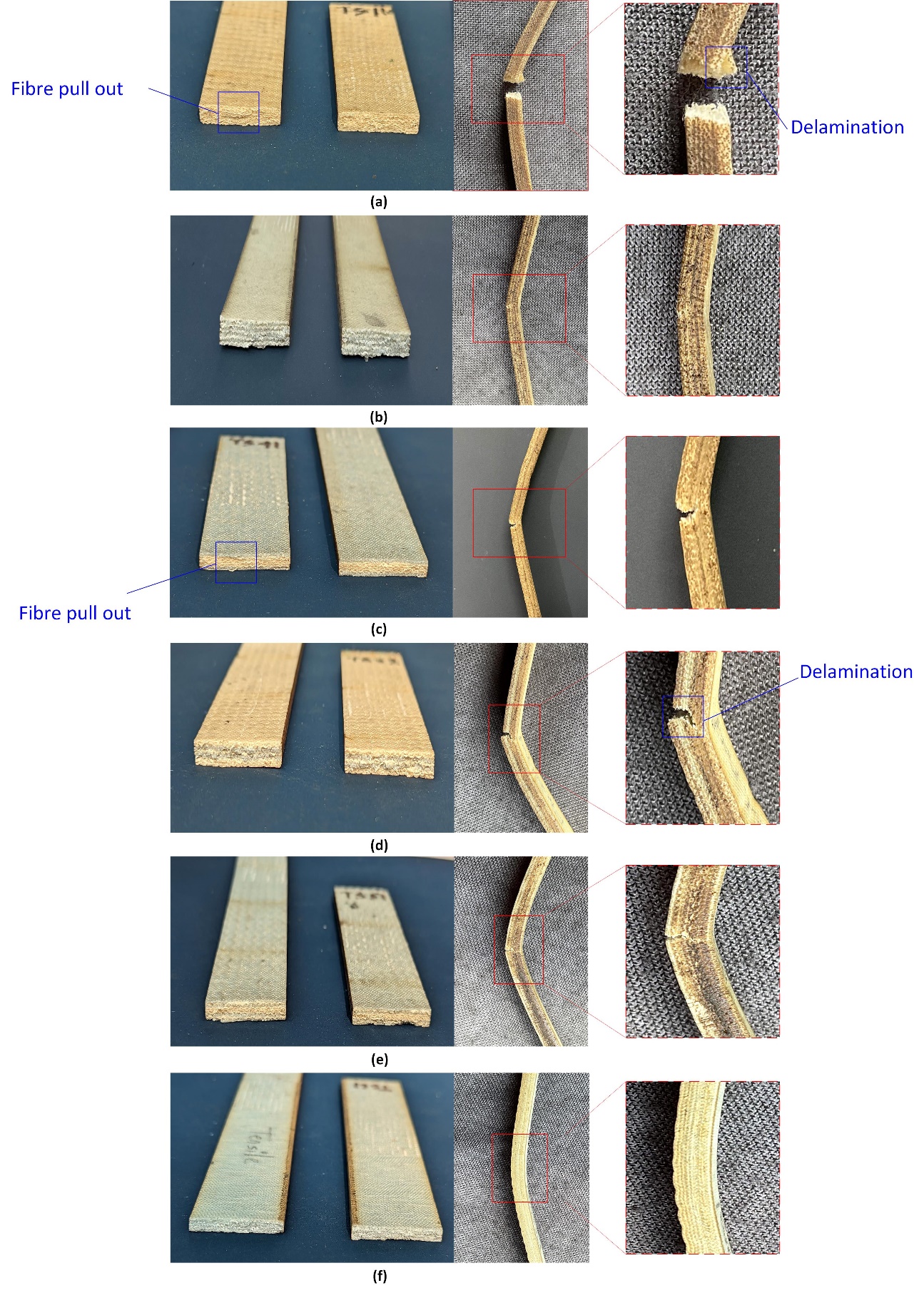


**Figure S1:** Tensile fracture of (a) J_9_ (b) J_1_P_1_J_1_P_1_J_1_P_1_J_1_P_1_J_1_ (c) J_2_P_2_J_1_J_2_P_2_ (d) P_2_J_5_P_2_ (e) P_1_J_1_P_1_J_3_P_1_J_1_P_1_ (f) P_9_
